# Post-operative Stereotactic Radiosurgery to Brain Metastases Cavity: A Large, Observational Single-Centre Series From the United Kingdom

**DOI:** 10.7759/cureus.89603

**Published:** 2025-08-08

**Authors:** Joanne Lewis, Caroline Dobeson, Akram Ali, Abdul Mian, Adam Young, Judith Mott

**Affiliations:** 1 Department of Clinical Oncology, Newcastle upon Tyne Hospitals National Health Service (NHS) Foundation Trust, Newcastle upon Tyne, GBR; 2 Department of Oncology, Royal Surrey County Hospital National Health Service (NHS) Foundation Trust, Guildford, GBR; 3 Department of Radiotherapy Physics, Newcastle upon Tyne Hospitals National Health Service (NHS) Foundation Trust, Newcastle upon Tyne, GBR

**Keywords:** brain metastases, cavity radiosurgery, overall survival, postoperative radiosurgery, stereotactic radiosurgery, stereotactic radiotherapy (srt)

## Abstract

Introduction

Stereotactic radiosurgery (SRS) is widely regarded as the standard of care after the resection of brain metastases in order to reduce local cavity recurrence risk. The objective of this study was to explore the reproducibility of published outcomes for patients receiving post-operative stereotactic radiosurgery (cavity SRS) in a National Health Service (NHS) setting for a non-selective series of patients. For our service, the median interval between surgery to cavity SRS (cSRS) is eight weeks, whereas similar timelines have been found to have a deleterious impact on survival in the published literature.

Materials and methods

This retrospective cohort study analysed outcomes for 100 consecutive cSRS patients treated between 2015 and 2019 at a Northern English cancer centre. A case note review was conducted, with collection of primary tumour, disease extent, SRS treatment details and outcome data.

Results

Median survival for all primaries was 16 months, with renal, melanoma and breast having optimum survival at 28, 26 and 17 months, respectively. Local relapse was seen in 6/100 patients, with a further 6/100 patients having leptomeningeal disease. Radionecrosis was rare (3/100). Dose prescription, size of PTV and number of metastatic sites did not produce a statistically significant impact on survival times. The detrimental impact of delay from surgery to SRS beyond 56 days reported by others was not evident in our series (p-value 0.786).

Conclusion

SRS to the surgical cavity after the resection of brain metastases in eligible patients produces favourable outcomes and demonstrates outcomes comparable to the world literature. Our study does not demonstrate a significant drop in survival with delay beyond eight weeks to cSRS, which may reflect a different cause of delay in our NHS setting than in other healthcare systems.

## Introduction

The incidence of brain metastases has risen largely as a consequence of developments in systemic therapy, in particular targeted molecular therapies and immunotherapy, which modify the natural history of tumours. Although cerebral metastases are a severe and life-threatening complication of cancer, options for treatment and patient prognosis have improved. The quality of life after brain metastasis treatment must be considered as a competing aspiration to the need for local control.

Following surgery for brain metastases, it has previously been established that whole brain radiotherapy reduces recurrence rates both in the cavity and elsewhere in the brain. Unfortunately, this approach is associated with considerable cognitive performance risk and resultant loss of quality of life [1,2]. Over the last decade, evidence has emerged to suggest that cavity SRS (cSRS) can reduce local recurrence post metastasectomy with a low risk of radionecrosis. The risk of recurrence in the local leptomeningeal region can be reduced by protocolised outlining in line with consensus guidance [3]. Studies comparing whole brain radiotherapy (WBRT) and cSRS have shown further evidence of desirable outcomes [4]. Local control rates at one year after cSRS are found to be in the region of 70-90% [4-6].

Tumour cavity SRS reduces recurrence risk compared to surgery alone and reproduces local control rates as equivalent to WBRT and with no survival disadvantage [6]. Recurrence elsewhere in the brain is more likely with cSRS compared to WBRT, but the impact of this is mitigated by MRI surveillance programmes [7]. Within the UK National Health Service (NHS), cSRS is currently only commissioned where there is evidence of residual disease or recurrence [8]. Since 2015, the stereotactic radiosurgery team at our cancer centre have had permission to proceed with a local programme of routine cSRS for all patients with resected brain metastases. This decision was made in response to published data suggesting enhanced local control with this approach. Published literature suggests that after cavity SRS, the probability of local control is improved from approximately 45-50% to 85% at one year [5,6].

The primary objective of this study was to measure survival outcomes in our patient cohort and examine factors affecting their survival. A secondary objective was to investigate whether the clinical outcomes in our service were comparable to those in the literature, despite the longer gap between surgery and cSRS than in other studies.

## Materials and methods

The first 100 consecutive patients to receive cSRS at our centre were treated between September 15, 2015 and October 17, 2019. Patient records were reviewed and treatment data collected for analysis. Each case was approved for cSRS following review at the SRS multi-disciplinary team meeting (MDT). The core membership of the MDT includes neuro-radiologists, neurosurgeons, clinical SRS oncologists, radiotherapy physicists, therapeutic specialised radiographers and the MDT coordinator. Criteria for approval included performance status (PS) 2 or better, absence of or treatable metastatic disease elsewhere, absence of surgical complications and a cavity that was felt suitable for fractionated or single fraction SRS. Patients may be unsuitable for cSRS due to performance status, disease extent, or changes in systemic disease. Assessment of co-existing intact cerebral metastases was also carried out to facilitate concomitant SRS treatment.

**Figure 1:**
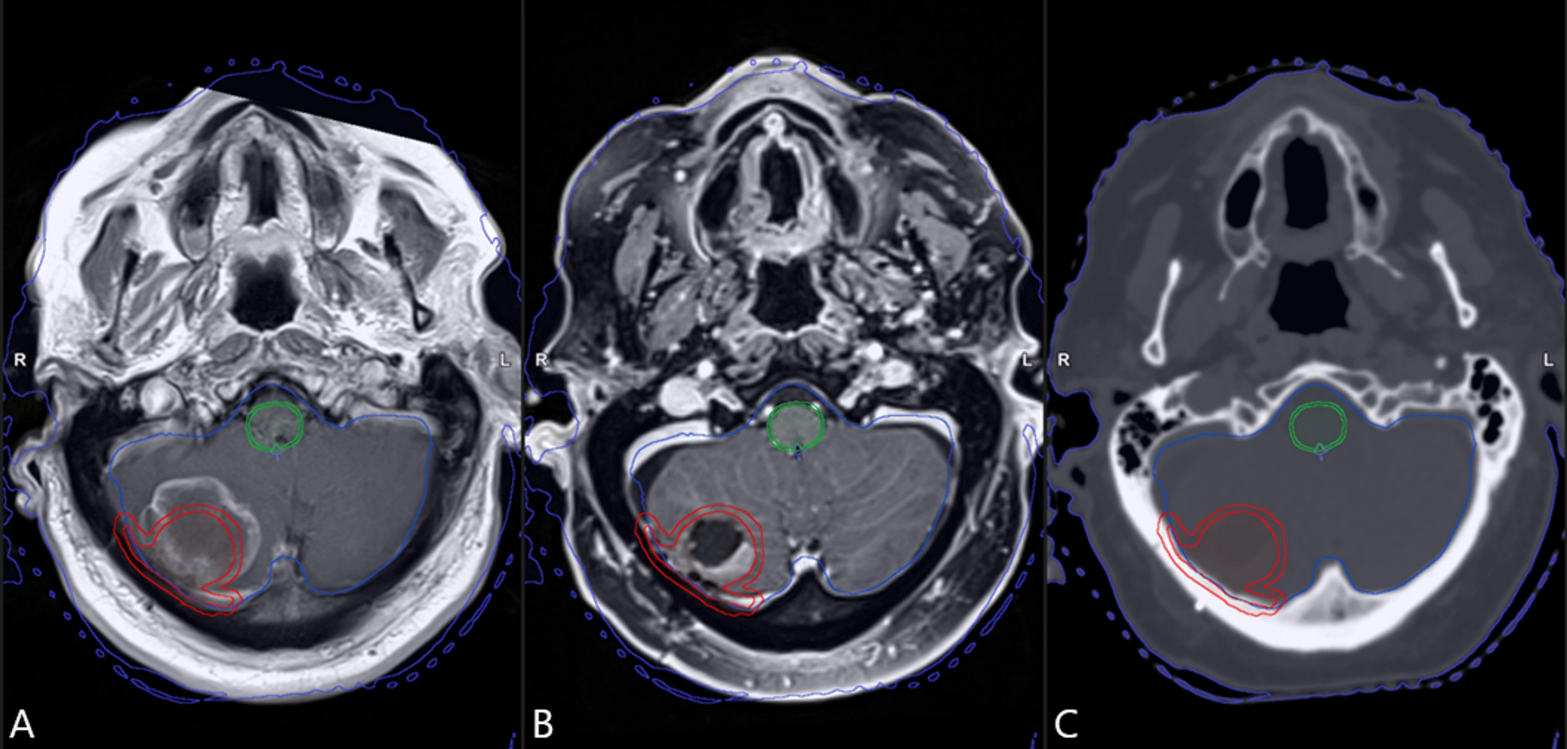
Pre- and post-operative imaging scans for a typical cavity SRS patient. (A) Pre-operative T1-weighted post-contrast diagnostic MRI scan, (B) post-operative T1-weighted 3D fast gradient echo sequence post-contrast planning MRI scan, (C) planning CT scan with 1 mm slice separation. In all the images, the tumour cavity outline (extended for pre-operative dural contact) and the planning target volume (PTV), grown with a 2 mm margin, are shown in red; the brainstem and planning organ at risk volume (PRV), grown with a 1 mm margin, are shown in green.

Following metastasectomy, a planning MRI scan was organised by the SRS team, usually within six weeks of surgery unless more time was needed for recovery from surgery. This scan was carried out using a 1 mm slice width volumetric sequence on a Siemens Espree 1.5T MRI scanner (Siemens Healthineers, Erlangen, Germany). Patients were fitted with a relocatable Brainlab (Munich, Germany) immobilisation mask and received a 1 mm slice thickness planning CT in the same sitting. Outlining of the tumour cavity was performed in accordance with the consensus guidelines for cavity SRS [3], with target volumes extended for 5 mm where the pre-resected metastasis had made dural contact. Figure 1 shows the pre-operative MRI scan for a typical cavity patient alongside the post-operative MRI and CT scans used for treatment planning.

**Figure 2:**
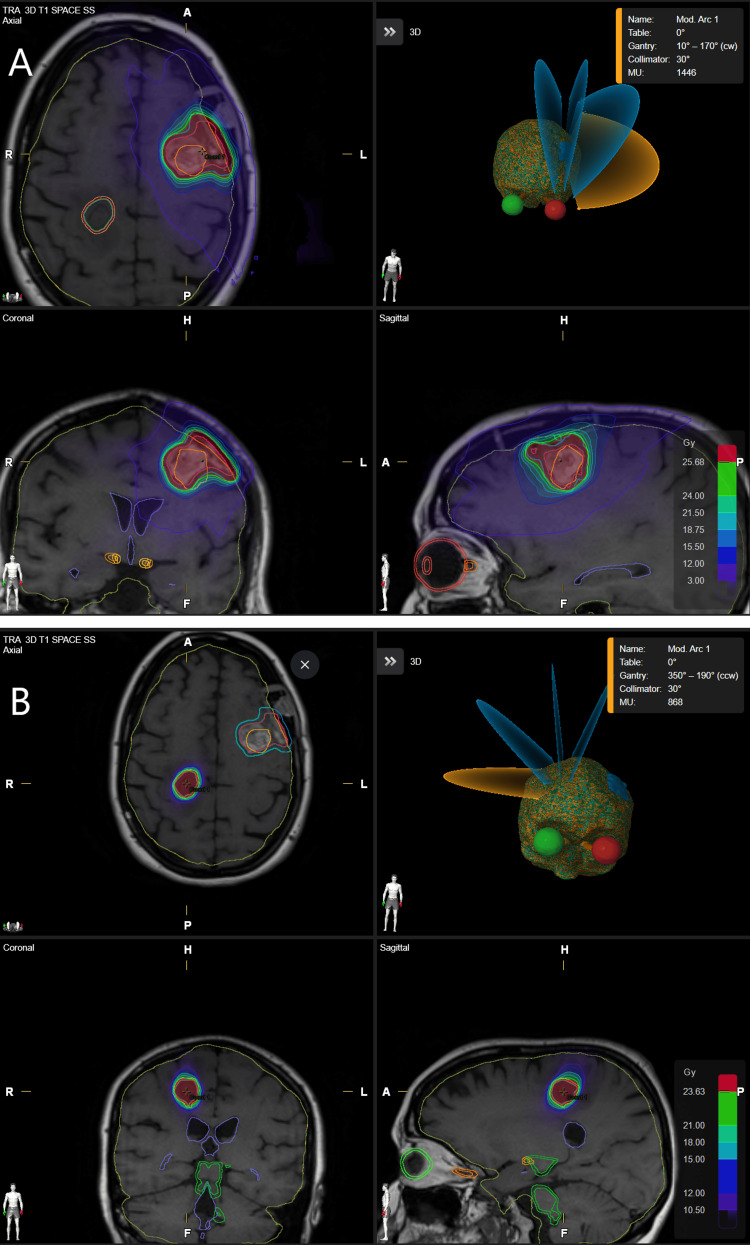
Dose distributions for a typical cSRS patient with a left-sided cavity and a right-sided intact metastasis, treated concurrently. (A) The left-sided cavity with the planning target volume (PTV) (blue) grown with a 2 mm margin from the clinical target volume (CTV) (red). The orange contour indicates the position of the lesion on the pre-operative MRI scans. The prescription isodose of 24 Gy in three fractions is shown in green. (B) The right-sided intact met with the PTV (red), grown with a 1 mm margin from the CTV (blue). The prescription isodose of 21 Gy in one fraction is shown in green. The intact met was treated concurrently with the cavity. cSRS: cavity stereotactic radiosurgery.

A 2 mm PTV margin was added to account for uncertainties in treatment planning or delivery. An appropriate dose prescription was selected based on cavity volume and tumour site, as well as consideration of any intact nearby lesions, which were treated concurrently where appropriate. Treatment planning software for the earlier patients was Brainlab iPlan, moving to Brainlab Elements during 2018 (Brainlab, Munich, Germany). Plans were created using multiple non-coplanar arcs delivered by either dynamic conformal arcs or a volumetric modulated arc therapy (VMAT) technique, prescribed to the 80% isodose or equivalent. Treatment was delivered on a stereotactic-enabled Varian Truebeam (Varian Medical Systems, Palo Alto, CA) with a 2.5 mm leaf width HD120 multi-leaf collimator (MLC) and ExacTrac patient positioning system (Brainlab, Munich, Germany). Figure 2 shows the dose distribution for a typical cSRS patient. The patient received 24 Gy in three fractions to the left-sided post-operative cavity, and 21 Gy in a single fraction to a right-sided intact met which was treated concurrently.

Each treatment (single or fractionated) was covered with dexamethasone prophylaxis to reduce acute post-ablative radiation oedema. The dose schedule for dexamethasone depended on whether the patient had single treatment or fractionated SRS and whether they were already taking steroid medication. Extensive data collection included information on primary staging and type, time elapsed from diagnosis and surgery, nature of post-SRS imaging outcomes, dose characteristics for SRS, cavity features and survival data. Statistical analyses were carried out using the IBM SPSS Statistics for Windows software package, version 27.0 (IBM Corp., Armonk, NY). Overall survival was estimated using the Kaplan-Meier method, with survival differences compared between groups using the Log-rank test. A p-value of <0.05 was considered statistically significant. Survival was calculated from the date of cSRS and patients who were event free were censored at the time of data collection.

## Results

The most common primary tumour group was non-small cell lung cancer (NSCLC) with 38% of cases. Breast cancer represented 20% of cases, and there was a lower proportion of melanoma, renal, gastro-intestinal tract (GIT), cancer of unknown primary (CUP), cervical, neuro-endocrine tumour (NET), small cell lung cancer, ovarian and tonsillar primary in descending order of frequency. Table 1 demonstrates the patient characteristics for the series.

**Table 1:** Patient characteristics for the 100 patients in the study cohort. n = number, M = male, F = female, cSRS = cavity stereotactic radiosurgery, NSCLC = non-small cell lung cancer, GIT = gastro-intestinal tract, f = fractions. Performance status (PS) was assigned using standard World Health Organization (WHO) criteria [9]; PS0 = able to carry out all normal activity without restriction, PS1 = restricted in strenuous activity but ambulatory and able to carry out light work, PS2 = ambulatory and capable of all self-care but unable to carry out any work activities; up and about more than 50% of waking hours.

Patient characteristics	Value	No. patients (total n=100)
Sex	M: F	45: 55
Age at cSRS (years)	30-39	2
	40-49	8
	50-59	23
	60-69	41
	70-79	25
	80-89	1
Performance status	0: 1: 2	19: 61: 20
Primary disease	NSCLC	38
	Breast	20
	Melanoma	12
	Renal	12
	GIT	10
	Other	8
Residuum post-surgery	Y: N	45: 55
Primary controlled at time of cSRS	Y: N	69: 31
Other intra-cranial lesions present at time of cSRS	Y: N	34: 66
Extra-cranial lesions present at time of cSRS	Y: N	49: 51
Dose prescription	15 Gy / 1 f	7
	18 Gy / 1 f	12
	21 Gy / 1 f	13
	24 Gy / 3 f	30
	25 Gy / 5 f	23
	27.5 Gy / 5 f	4
	30 Gy / 5 f	3
	30 Gy / 6 f	8

**Figure 3:**
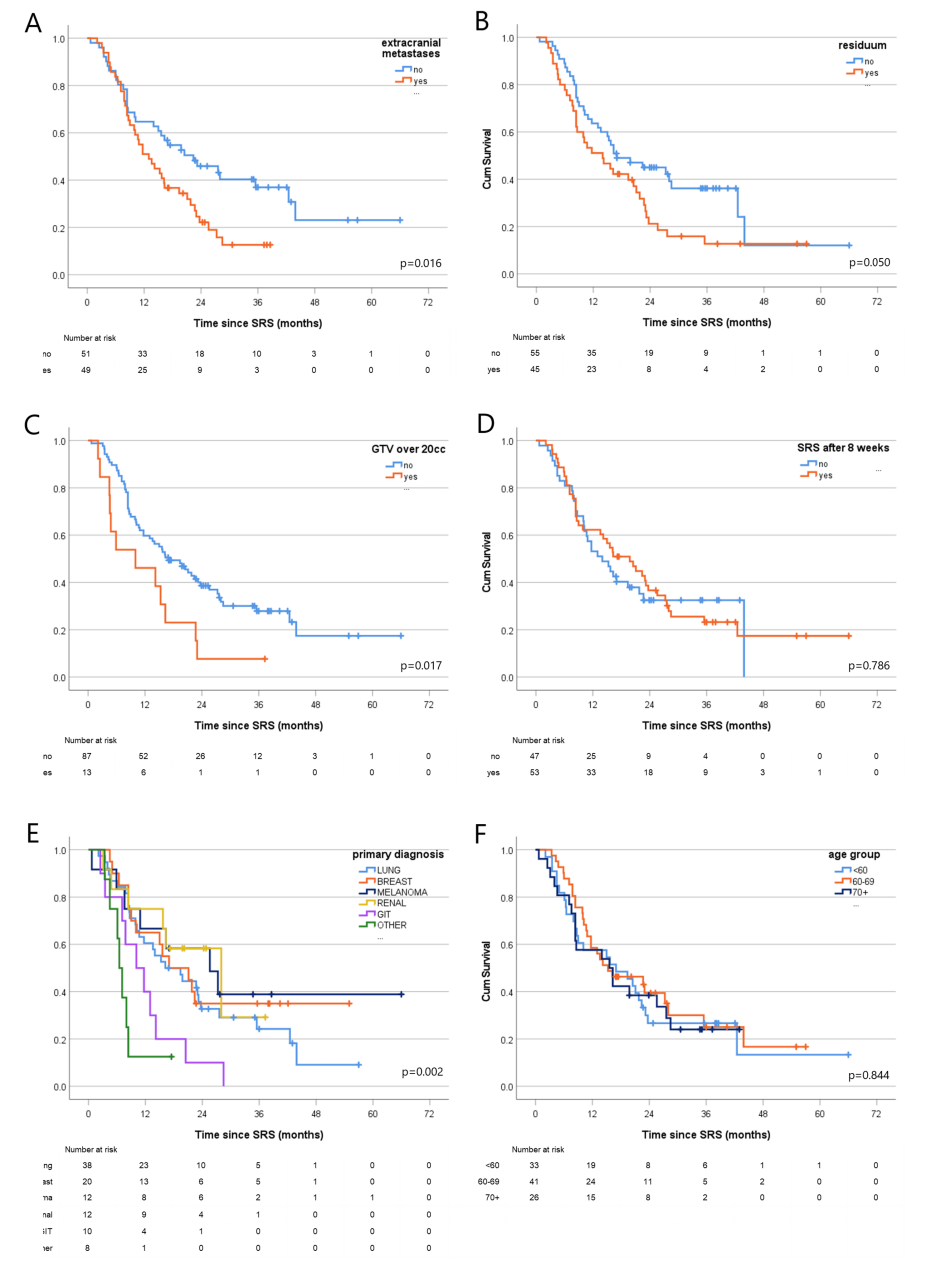
Overall survival curves for different patient characteristics: (A) presence of extracranial metastases; (B) post-surgery residuum; (C) GTV size over 20 cc; (D) time to cSRS after surgery of greater than eight weeks; (E) primary diagnosis; (F) age group. GTV = gross tumour volume, cSRS = cavity stereotactic radiosurgery.

Figure 3 demonstrates Kaplan-Meier survival graphs for patients according to primary, extra-cranial disease presence, gross tumour volume (GTV) size, residuum (defined by radiologist suspicion on post-operative MRI scans), time to cSRS and age.

Median patient follow-up was 16 months (33 months for surviving patients). The median overall survival (OS) was 16 months (95% confidence interval (CI) 10-21). There was a statistically significant difference in OS between patients whose primary diagnosis was one of the common primary sites (breast, NSCLC, melanoma, renal) (20 months, 95% CI 14-26) and other primary diagnoses (8 months, 95% CI 6-10), p < 0.001. The best median survival was seen in renal patients with 28 months, melanoma, breast, NSCLC, gastro-intestinal tract (GIT) and others had median survival of 26, 17, 16, 10 and 7 months, respectively (Figure 3E). Table 2 shows the percentage of patients in each primary group alive at one and two years after cSRS.

**Table 2:** Overall survival after cavity SRS for the 100 patients in the study cohort. NSCLC = non-small cell lung cancer, GIT = gastro-intestinal tract, SRS = stereotactic radiosurgery.

Primary	Median overall survival (months)	95% confidence interval (months)	1 year survival	2 year survival
NSCLC	16	8–25	61%	33%
Breast	17	5–29	65%	35%
Melanoma	26	9–42	67%	58%
Renal	28	11–45	75%	58%
GIT	10	4–16	40%	10%
Other	7	5–8	13%	0%

Almost a third of the patients (n=30) had treatment of other intact metastases at the same time as cSRS; the presence of other synchronous brain metastases treated with SRS did not significantly impact on median survival time (p=0.623). Median OS was 13 months (95% CI 7-19) for patients with synchronously treated intact metastases versus 16 months (95% CI 10-23) for patients where only the cavity was treated.

Other parameters not affecting survival significantly were age, sex, performance status at cSRS, and a delay of over 56 days to treatment (p=0.786). The only planning criteria that produced statistically significant results was a GTV size above 20 cc; for the 13% of those larger cavities survival appeared to be adversely impacted with a median survival of 10 months (95% CI 0-21) versus 17 months (95% CI 11-23) for those with smaller cavities (p=0.017). It is important to note that over a quarter of the patients were elderly (>70); their median survival was equivalent to younger age groups (Figure 3F).

**Figure 4:**
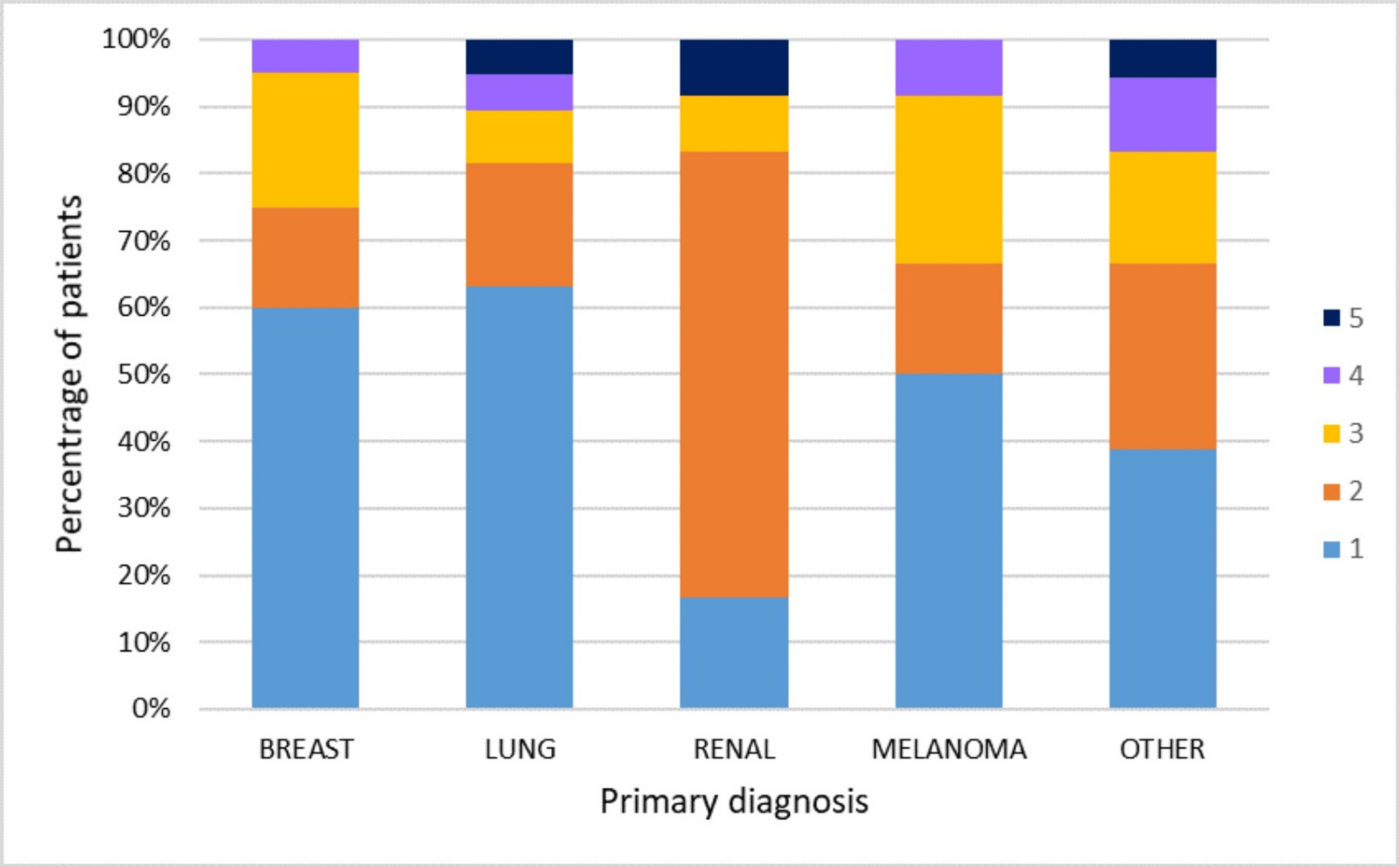
Number of sites of metastatic disease for patients with each primary diagnosis. Primary diagnosis of the 100 patients in the study cohort was breast (n = 20), lung (n = 38), renal (n = 12), melanoma (n = 12) or other (n = 18). Patients with only one site are those whose metastatic disease is confined to the brain.

There was a significant difference in survival post-cSRS for patients with extracranial metastatic disease compared to patients where the brain was the sole site of metastatic relapse (median survival 13 months (95% CI 8-18) versus 22 months (95% CI 11-34), p=0.016, see Figure 3A). When the number of metastatic sites was compared between primary groups, the renal primary patients had significantly more metastatic sites at the time of treatment of brain metastases compared to the other groups. Less than 20% of renal primary patients had disease confined to the brain, compared to over 50% of NSCLC, breast or melanoma primary patients, p-value 0.014 using Fisher's Exact Test (Figure 4).

At the time of cSRS, 69% of patients had good control of their primary disease. The impact on survival of non-controlled primary disease was not found to significantly influence survival (p=0.344), although there was a trend for reduced survival to a median of 12 months (95% CI 6-18) compared to 16 months (95% CI 11-22) if active primary disease was present.

**Figure 5:**
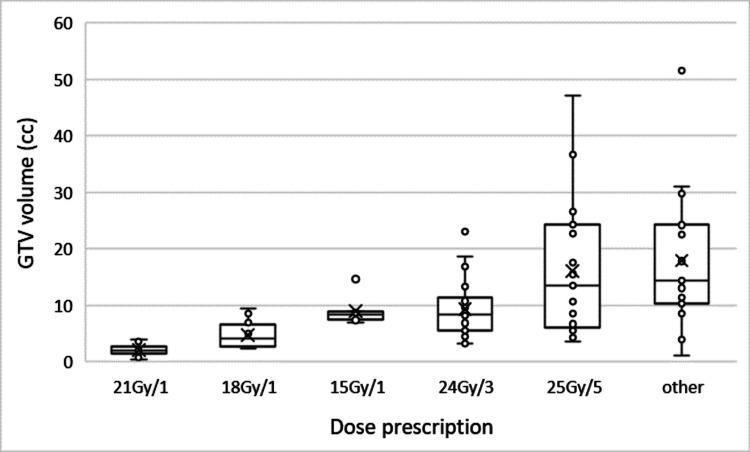
The relationship between GTV volume and dose prescription for cavity SRS. GTV = gross tumour volume, SRS = cavity stereotactic radiosurgery.

The dose was prescribed to 80% isodose equivalence in all cases. The most prescribed dose was 24 Gy in three fractions and 25 Gy in five fractions (Table 1). Other doses used included 15/18/21 Gy in a single fraction, 27.5 Gy in five fractions and 30 Gy in six fractions. The relationship of dose fractionation to GTV size is demonstrated in Figure 5.

Post-operative residuum appeared to impair survival (p=0.05); patients with evidence of residual disease had a median survival of 14 months (95% CI 7-21) compared to 17 months (95% CI 6-28) for those with a clear cavity. 12% of patients either developed local recurrence after cSRS (n=6) or leptomeningeal (LM) relapse (n=6) associated with the cavity; these occurrences were radiologically diagnosed. The mean interval to developing recurrence was 11 months, which was similar to the mean interval of 13 months for the 3% that developed radionecrosis. Radionecrosis was pathologically proven in one patient who had surgery, whilst the other two diagnoses were based on radiological appearance. There was no association between local recurrence and residual disease status pre-cSRS. Thirty-one patients had further treatment to distant lesions; eight patients received WBRT, eight had additional surgery, and 19 had further courses of SRS.

## Discussion

Optimum control of cerebral metastatic disease significantly contributes to the care of the cancer patient. Due to the progress of targeted and immunotherapies, there are now opportunities for increased survival if therapeutic windows and patient fitness can be improved. As it is also a priority to maintain cognitive performance, cSRS is a critical tool to achieve maximal length and quality of life (QoL) in those who have required surgery for cerebral metastases, whilst facilitating the avoidance of whole brain radiotherapy.

Published series quote median survival time to be between 11-14 months in patients given post-operative cSRS [5,10]. There was no survival advantage when WBRT was given instead of SRS in Mahajan et al.’s 2017 MD Anderson Cancer Center series [11] or in Lamba et al.’s systematic review [5]; however, there was a significant local control advantage when cSRS was compared to observation alone.

In addition, the N107 study demonstrated improved cognitive performance scale and QoL when SRS was compared to WBRT [12]. In the 2012 Stanford series by Choi et al., only 28% of patients ultimately went on to receive WBRT, the local cavity control rate at one year was 90.5%, and distant brain failure was 46% [13]. In our series local control rate at 16 months was 88% and 31% patients had further treatment for distant lesions, with 19% receiving additional SRS. This complements the Stanford findings, considering some with distant failure wouldn’t have been candidates for further SRS. Discovering brain lesions at an early stage with MRI surveillance is likely to offer the opportunity for further SRS and contribute to enhanced survival chances. It is the authors’ view that advocating SRS for cavity is not justified unless there is a commitment to follow-up MRI surveillance.

In our series, the median survival time was found to be 16 months. The median for renal was 28 months, melanoma 26 months, breast 17 months and NSCLC 16 months. The survival curves for these four primary sites are similar until two years, at which point the melanoma and renal cohorts demonstrate a survival advantage, perhaps in keeping with the recent enhanced effectiveness of systemic options in this group of patients. The median survival times of our cavity SRS cohort are consistent with worldwide published series and hence validate the selection criteria for both surgery and cavity SRS within our local practice.

It is interesting to consider why the renal group of patients are outliers to the rest of the cohort in terms of presenting with a greater number of metastatic sites than other primaries. This may suggest that brain involvement is a relatively late event in the natural history of the disease compared to lung or breast cancer patients. It may also be an indicator of the impact of tyrosine kinase inhibitor therapy transforming the natural history of renal cancer and the variable extent to which these molecules can penetrate the blood-brain barrier to afford brain protection.

Prior to 2018, patients referred to our service from other Trusts in the region were sometimes missing time points for post-surgical MRI imaging due to poor availability in outlying hospitals. Following an operational policy review, post-operative MRI imaging was brought into our own reserved appointment system; this change reduced the median time from surgery to cSRS. We investigated whether a reduced time to cSRS conferred a survival advantage, but found no statistical significance associated with a delay of more than 56 days (p=0.786) (Figure 3D).

This contrasts with the evidence reviewed by Marchan et al., Roth O’Brien et al. and Yusuf et al. in their publications [10,14,15]. In particular, the Roth O’Brien study looked at the real-world impact of delays beyond four weeks and concluded that delays of beyond eight weeks were the equivalent of no treatment at all [14]. This may be a reflection of the reasons for delay in the UK being more likely to be operational factors, and in the USA, more likely to be disease and hence prognostic factors. For example, Yusuf et al. found that the longest delays were due to the need for rehabilitation following surgery [15]. It is important, therefore, to report that the finding that the median interval from surgery to cSRS at 56 days (mean 64 days) was not found to adversely affect outcome in our patient cohort. We have found that operational delay beyond eight weeks is not a contraindication to proceeding with effective treatment and recognise we are the first to suggest this finding.

There is emerging evidence that for larger cavities, fractionated SRS results in equivalent or better local control than single (often compromised) fraction treatments with less toxicity [16-18]. The International Stereotactic Radiosurgery Society (ISRS) suggested that a biologically equivalent dosage above 1 x 15 Gy is required for optimal control of cavities [19]. Their recommendation of 24 Gy in three fractions or 30 Gy in six fractions for larger volumes would theoretically result in better local control rates [18]. In our series, we have a significant number of fractionated SRS patients with larger cavities facilitated by our relocatable frame system; we could not demonstrate a significant survival impact between the different dosage schedules, consistent with the referenced evidence.

Recurrence rates, including leptomeningeal disease (LMD), are comparable to other series. LMD seeding in a nodular pattern is recognised as a risk of surgery for brain metastases. The consensus guidance for cSRS recommends defining an elongated target volume at sites of pre-resection meningeal contact. Future studies are planned to investigate if LM relapse rates can be reduced with the concept of pre-operative SRS, following on from encouraging retrospective series evaluations [20,21].

The limitations of this study are few, other than its retrospective nature and univariate analysis. The study focuses on overall survival as this data was available for all patients and could be independently verified. As with any retrospective study of this nature, there were limitations in the completeness of imaging follow-up, which was performed at the clinician's discretion based on factors such as systemic disease status. It was therefore not possible to analyse local control with the same level of certainty. However, as local control is one of the main factors affecting overall survival, we are reassured that our median survival data is comparable with other published data series.

As this was a continuous cohort of all patients given cSRS in a regional centre, our findings are not susceptible to selection bias; our criteria for proceeding to treatment are clear and accepted as standard by the SRS community. The main outcomes of interest in our series were the favourable survival times and the lack of impact of delay to SRS compared to accepted previous published trends. These findings are based on hard, robustly recorded timepoints and therefore should not be subject to error. It is also important to note that we have calculated survival from the date of surgery, whereas some other published series record survival from an earlier time point. As such, our reported survival may represent a slight understatement compared to other published literature.

A further important point is that the size of this study cohort satisfies the criteria for inclusion in meta-analyses used in relevant review papers; a retrospective series of at least 100 patients and a prospective series, minimum of 30 [19,22]. The total number in the analysed cohorts involved 1187 patients, and only seven retrospective series satisfied the rule, adding credibility to this series, the only one in a UK-based population of patients to involve such a substantial number of patients. A comparison of outcomes from this study to other published series is shown in Table 3.

**Table 3:** Summary of the literature pertaining to clinical outcomes after post-surgical cavity SRS. R = retrospective, Ph2 = Phase 2, LC = local control, LM = leptomeningeal, NR = not recorded, OS = overall survival, f = fractions, LINAC = linear accelerator, SRS = stereotactic radiosurgery.

Name	Data type	Number of patients	Technique	LC rate %	Median follow-up (months)	Rate of LM disease %	Median OS (months)	Radionecrosis rate (%)	Radiotherapy prescription
Atalar et al. (2013) [23]	R	165	Cyberknife	87	12	13	17	NR	Various
Luther et al. (2013) [24]	R	120	Gamma Knife	86	8	NR	17	NR	27 Gy / 3 f
Minniti et al. (2013) [25]	R	101	LINAC	91	16	NR	17	9	27 Gy / 3 f
Brennan et al. (2014) [26]	Ph2	39	LINAC	85	12	NR	15	17	18 Gy / 1 f
Iorio-Morin et al. (2014) [27]	R	110	Gamma Knife	75	10	11	11	22	18 Gy / 1 f
Keller et al. (2017) [28]	R	181	LINAC	86	12	14	17	18	33 Gy / 3 f
Combs et al. (2018) [29]	R	181	LINAC	80	12	NR	16	4	35 Gy / 7 f
Current study	R	100	LINAC	88	16	6	16	3	Various

Future studies will help to resolve uncertainties in the optimum sequencing of SRS in resected brain metastases. The randomised pre- vs. post-operative SRS study by MD Anderson Cancer Center that is currently recruiting will define if pre-op SRS reduces the incidence of LM spread and radionecrosis [30]. The Saturnus Study, based in Munich, is in recruitment and is the first Phase 3 randomised research comparing single fraction versus hypofractionated schedules with comparison of local control chance and toxicity [31]. Results are expected by late 2025.

Yet to be addressed is the understanding of whether cSRS remains necessary in completely resected patients who have targeted therapy or immunotherapy options, such as those with melanoma or epidermal growth factor receptor (EGFR)-positive NSCLC. Such niche questions may be difficult to further investigate in a controlled trial setting.

## Conclusions

The survival outcomes of our patient cohort are consistent with published literature. The results could be applicable to the wider UK patient population, offering the potential for all patients to benefit from routine cSRS regardless of resection status. An unexpected and original finding of our large series is that a delay to cSRS of beyond eight weeks did not correlate with worse survival. This may be explained by the underlying reason for delay differing between healthcare economies, but is worthy of further study.
